# Effect of different surface treatments on shear bond strength of ceramic brackets to old composite

**DOI:** 10.1186/s40824-020-00199-y

**Published:** 2020-11-25

**Authors:** Homa Farhadifard, Loghman Rezaei-Soufi, Maryam Farhadian, Parisa Shokouhi

**Affiliations:** 1 Department of Orthodontics, School of Dentistry, Hamadan University of Medical Sciences, Hamadan, Iran; 2 Department of Restorative Dentistry, School of Dentistry, Dental Research Center, Hamadan University of Medical Sciences, Hamadan, Iran; 3 Department of Biostatistics, School of Public Health and Research Center for Health Sciences, Hamadan University of Medical Sciences, Hamadan, Iran; 4 School of Dentistry, Hamadan University of Medical Sciences, 6517838677 Shahid Fahmideh Street, Hamadan City, Hamadan, Iran

**Keywords:** Shear strength, Er,Cr:YSGG laser, Sandblasting, Grinding, In vitro

## Abstract

**Background:**

At present, the demand for orthodontic treatment is on the rise. On the other hand, evidence shows that the bond strength of composite resins to old composite restorations is often unreliable. Therefore, the aim of this in vitro study was to assess the effect of different surface treatments on shear bond strength (SBS) of ceramic brackets to old composite restorations.

**Methods:**

In this in vitro experimental study, 60 nano-hybrid composite discs were fabricated. For aging, the discs were incubated in deionized water at 37 °C for 1 month. Next, they underwent 4 different surface treatments namely acid etching with 37% phosphoric acid, sandblasting, grinding, and Er,Cr:YSGG laser irradiation. Ceramic brackets were then bonded to the discs and underwent SBS testing.

**Results:**

The maximum mean SBS value was obtained in the grinding group (9.16 ± 2.49 MPa), followed by the sandblasting (8.13 ± 2.58 MPa) and laser (6.57 ± 1.45 MPa) groups. The minimum mean SBS value was noted in the control group (5.07 ± 2.14 MPa).

**Conclusion:**

All groups except for the control group showed clinically acceptable SBS. Therefore, grinding, sandblasting, and Er,Cr:YSGG laser are suggested as effective surface treatments for bonding of ceramic orthodontic brackets to aged composite.

## Background

The increasing demand of adults for orthodontic treatment has been associated with some problems. Since many adult patients requiring orthodontic treatment have several composite, amalgam, and porcelain restorations, orthodontists face more challenges in bracket bonding to restored teeth. This problem is more evident in patients with composite restorations especially in the labial surface of their maxillary incisors or buccal surface of their posterior teeth [1].

Evidence shows that the bond strength of composite resin to old composite restorations is often unreliable [2]. As composite restorations age, the number of available vinyls for cross-polymerization to the new composite layer decreases; therefore, chemical bonding between the old composite and the new composite resin is challenging [3]. It has been proven that the etching process with orthophosphoric acid cannot alter the surface topography of composite resin, and only cleans the superficial layer [4, 5] Therefore, increasing the bond strength between the new and old composite usually requires additional surface roughening of the old composite to improve mechanical interlocking and subsequent coating of the surface with the bonding agent to improve surface wetting and chemical bonding [6]. According to previous studies [7–9], mechanical cross-linking is the most important factor affecting the bond strength of old to new composite.

Replacement of loose brackets is a time-consuming and costly procedure. Therefore, enhancing the bond strength is a priority in orthodontic treatment [10]. Controversy exists regarding the most suitable composite surface treatment to ensure optimal bond strength [3, 6, 11, 12]. Some previous studies [1, 13, 14] recommended surface roughening by bur; while, others [5, 6, 15, 16] considered sandblasting as the best surface treatment.

On the other hand, some recent studies evaluated the efficacy of erbium family of lasers (Er:YAG and Er,Cr:YSGG) for etching of dentin and enamel, composite surface roughening for enhancement of bond strength, and bracket base reconditioning. Considering the effective role of erbium lasers for surface roughening and increasing the micromechanical retention, it seems that Er,Cr:YSGG laser can be used for surface conditioning to increase the bond strength of orthodontic brackets to old composite [17, 18].

On the other hand, ceramic brackets were introduced to meet the increasing esthetic needs of orthodontic patients [14]. These brackets are made of aluminum oxide and are available in two types: polycrystalline alumina and mono-crystalline alumina [13]. They have advantages such as optimal biocompatibility, favorable esthetics, high heat resistance, and suitable chemical stability [14]. Also, the bond strength of ceramic brackets to the enamel is usually higher than that of stainless steel brackets [13].

Considering the increasing demand of orthodontic patients to use ceramic brackets due to their optimal esthetics, and the importance of adequate bond strength of brackets to old composite, the aim of this study was to assess the effect of different surface treatments on shear bond strength (SBS) of ceramic brackets to old composite restorations.

## Methods

In this in vitro experimental study, 60 composite discs (6 mm in diameter and 4 mm in height) were fabricated from a nano-hybrid composite resin (Filtek™Z250; 3 M ESPE®, St. Paul, USA) by using plastic molds measuring 4 mm × 6 mm. The composite was applied into the mold in two layers, and each layer was light-cured (Woodpecker, China) with a light intensity of 850 mW/cm^2^ for 20 s.

The surface of all composite specimens was polished with coarse, medium, fine and extra-fine polishing discs (FGM®; Diamond Pro, Marca, Brazil) in an orderly manner. A contra-angle low-speed handpiece was used for polishing with short, intermittent movements (3 times for each disc). After using each disc, the surface of specimens was rinsed with water for 10 s and dried with oil-free compressed air. Afterwards, the specimens with voids on their surface or deformities were excluded and replaced. In order to expedite the aging process of composite, the specimens were incubated at 37 °C for 1 month in deionized water [19]. For easier handling, the specimens were mounted in acrylic resin blocks (Acropars 200, Marlik Medical Industries Co., Tehran, Iran) such that the composite surface was parallel to the debonding blade [16]. The specimens were then randomly divided into four groups (*n* = 15):

Group 1 (control): The surface of the discs was etched by applying a thin layer of 37% phosphoric acid (DenFil® Etchant-37; Vericom, Korea) for 60 s, washed with water for 60 s, and dried with compressed oil-free air [1, 13, 15, 20].

Group 2 (sandblasting): The sandblasting process was performed by a micro-etcher (GD Carlo de Giorgi Sri, Italy), using 50 μm aluminum oxide particles at 65 psi pressure for 7 s. The distance between the tip of the micro-etcher and the disc surface during sandblasting was 10 mm [20, 21].. Group3 (grinding): Abrasion was accomplished using a tapered diamond bur (G847, 016, D8, Dia.Tessin®, Vanetti, Switzerland) with a high-speed hand-piece (BienAir BORA®, Bienne, Switzerland) in two directions perpendicular to each other, under continuous water spray. To ensure uniform pressure application on the specimens, the grinding process of all specimens was performed by one operator. The rotating bur was used three times on each composite surface [16].

Group 4 (laser): Er,Cr:YSGG laser with 2780 nm wavelength, 3.5 W power, 20 Hz frequency, 80% water and 60% air in H mode was irradiated using a gold handpiece with MZ6 tip with 600 μm diameter for 20 s and from 2 mm distance (non-contact mode) from the surface [22].

### Bonding process

Ceramic brackets (Dentsply GAC International, NY, USA) were bonded to the surface of composite discs using an orthodontic adhesive. A thin layer of adhesive primer (Transbond XT; 3 M Unitek®, Monrovia, CA, USA) was applied on the surface in all groups that had already been etched with 37% phosphoric acid. Adhesive paste primer (Transbond XT, 3 M Unitek®, Monrovia, CA, USA) was applied on the base of the bracket, and the bracket was centered on the disc surface. While adjusting the bracket, a constant pressure was applied to minimize the resin thickness. A dental explorer was used to remove excess resin around the brackets. The adhesive was then light-cured (Woodpecker, China), with a light intensity of 850 mW/cm^2^ through the bracket for 5 s. All specimens were stored in distilled water at 37 °C for 48 h prior to the SBS test.

### Bond strength test

**Fig. 1:**
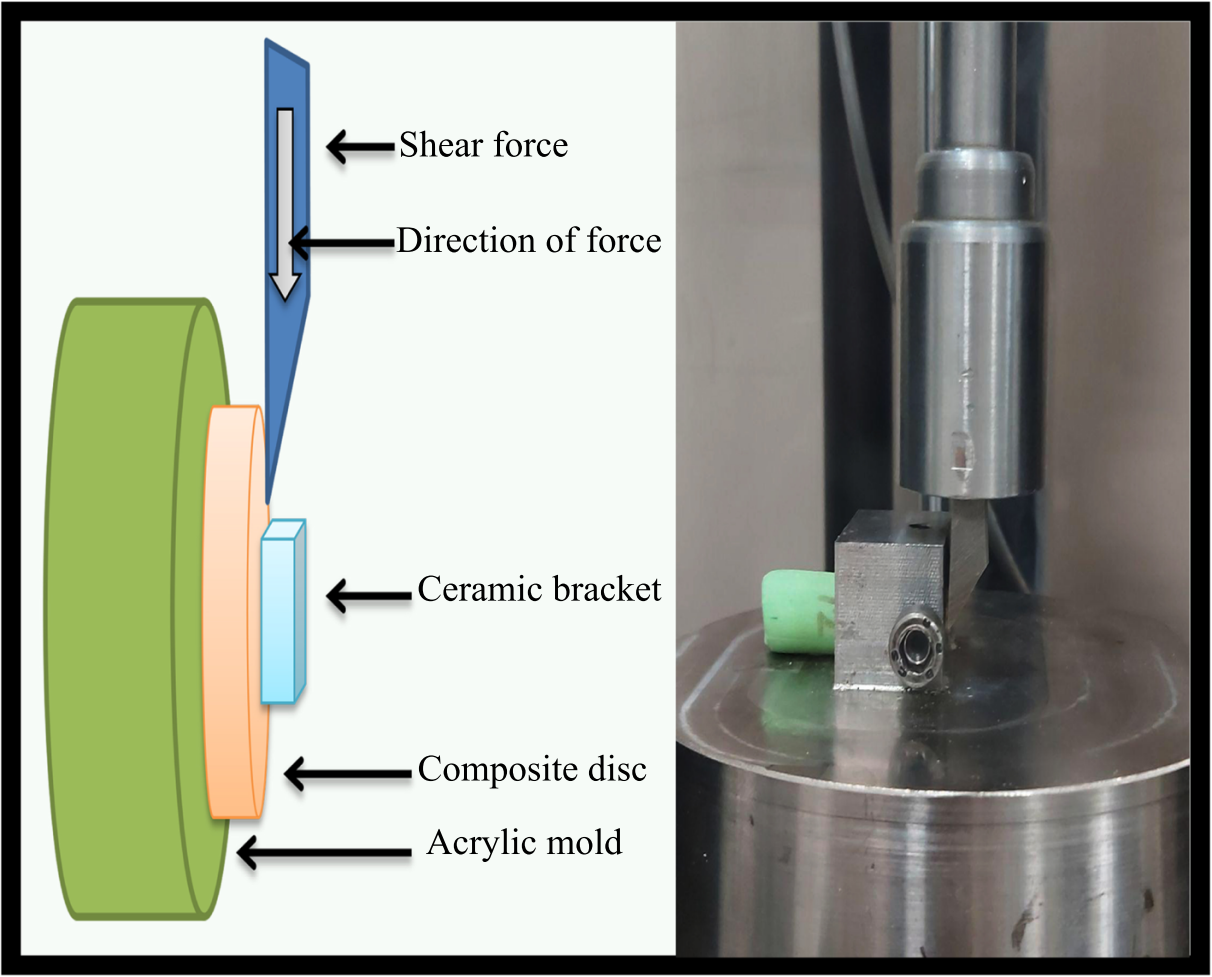
Samples in the process of applying shear force to the base of the bracket in SBS test

The SBS test was performed by a universal testing machine (STM-20; Santam®, Tehran, Iran). Shear force was applied by a one-sided cutting blade to the base of the bracket and parallel to the composite resin/adhesive/bracket interface at a crosshead speed of 0.5 mm/min. The force required for bracket debonding was recorded in Newtons (Fig. 1). The obtained values were converted to megapascals (MPa) using the following equation:

Shear bond strength = F/A (N/mm2 or MPa), where F is the debonding force in Newtons, and A is the cross-sectional area of the bracket base in millimeters.

After the SBS test, the brackets and composite discs were analyzed under a stereomicroscope at × 10 magnification in order to assess the mode of failure. The adhesive remnant index (ARI) was also determined to specify the location of bond failure in the composite surface, adhesive surface, or the bracket base. According to Artun and Bergland [14], the ARI scores ranged from 0 to 3: Zero indicated lack of adhesive on the disc surface, 1 indicated less than half of the adhesive left on the disc surface, 2 indicated more than half of the adhesive left on the disc surface, and 3 indicated the entire adhesive left on the disc surface.

Fracture of the composite disc following the application of shear force was also investigated under the microscope.

### Scanning electron microscope

In order to evaluate the micro-topography of composite surfaces after undergoing different surface preparations, two specimens from each group were randomly chosen. Afterwards, the surface of the specimens were coated with a layer of gold and were examined using scanning electron microscope (FEI ESEM, QUANTA 200, USA) under 500× magnification.

### Statistical analysis

The data were analyzed using SPSS version 21. The mean, standard deviation, minimum and maximum SBS values were calculated for the study groups. Due to the normal distribution of the SBS data in the groups, one-way ANOVA was used to determine the most effective surface treatment method, followed by the Tukey’s post-hoc test for pairwise comparisons. To compare the ARI scores among the four groups, the Kruskal-Wallis test was applied followed by the Bonferroni adjustment for multiple tests. The level of significance for all tests was set at 0.05 (*P* < 0.05).

## Results

Table 1 shows the mean and standard deviation of SBS of the groups. The highest mean SBS was noted in the grinding group while the lowest mean SBS value was recorded in the control group. Based on one-way ANOVA, the difference in the mean SBS was significant among the groups (*P* = 0.002). Thus, pairwise comparisons were performed by the Tukey’s post-hoc test. According to the results, the difference in the mean SBS was significant between the control and grinding groups (*P* = 0.000), the control and sandblasting groups (*P* = 0.002), and also the grinding and laser groups (*P* = 0.012). There was no significant difference in the mean SBS of the control and laser groups (*P* = 0.257), grinding and sandblasting groups (*P* = 0.577), and laser and sandblasting groups (*P* = 0.231).

**Table 1:** Mean, standard deviation, minimum and maximum SBS (MPa) values in the four groups (*n* = 15)

Group	Minimum	Maximum	Mean ± Std. Deviation
Control	2.51	8.97	5.07 ± 2.14
Grinding	4.23	12.34	9.16 ± 2.49
Sandblasting	4.87	13.49	8.13 ± 2.58
Er,Cr:YSGG laser	4.23	10.34	6.57 ± 1.45

Table 2 shows the frequency of modes of failure (ARI scores) in the study groups. The ARI score of 0 was most commonly recorded in the control group while the ARI score of 1 was dominantly seen in the laser group. The grinding group followed by the sandblasting group had the highest frequency of ARI score of 3. The Kruskal Wallis test showed that there was a significant difference in bond failure modes among the groups (*P* < 0.001).

**Table 2:** Frequency of ARI scores in the study groups

ARI score		Groups				Total
		Control	Grinding	Laser	Sandblasting	
0	-Number -% in group	9 60%	1 6.7%	2 13.3%	1 6.7%	13 21.7%
1	-Number -% in group	5 33.3%	1 6.7%	9 60%	2 13.3%	17 28.3%
2	-Number -% in group	1 6.7%	3 20%	3 20%	1 6.7%	8 13.3%
3	-Number -% in group	0 0%	10 66.7%	1 6.7%	11 73.3%	22 36.7%
Total	-Number -% in group	15 100%	15 100%	15 100%	15 100%	60 100%

The results of the Mann-Whitney test with Bonferroni adjustment showed that the difference in ARI scores was significant between the control and grinding (*P* < 0.001), control and sandblasting (*P* < 0.001), and laser and sandblasting (*P* = 0.001) groups (Table 3). It should be noted that for this test, *P* value < 0.006 was considered statistically significant.

**Table 3:** Pairwise comparisons of ARI score in groups by the Mann-Whitney test and Bonferroni adjustment

Groups	*P*-value
Control and grinding	< 0.001
Control and laser	0.008
Control and sandblasting	< 0.001
Grinding and laser	0.001
Grinding and sandblasting	0.817
Laser and sandblasting	0.001

## Discussion

The present study was conducted to evaluate the effect of four surface treatments on SBS of ceramic orthodontic brackets to aged nano-hybrid composite in vitro. According to our findings, grinding and sandblasting resulted in higher SBS values in comparison to the control group. Meanwhile, Er,Cr:YSGG laser application did not result in a significantly higher SBS value, compared with the control group.

**Fig. 2:**
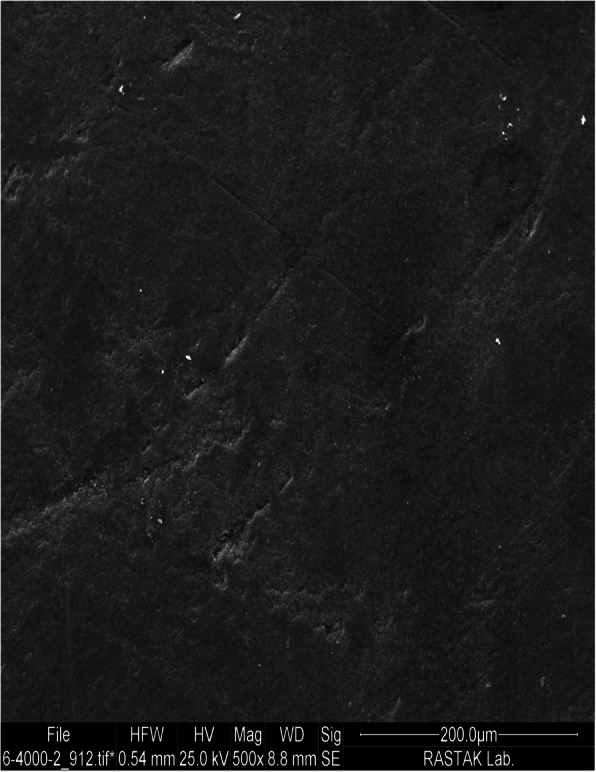
SEM micrograph of composite surface treated with 37% phosphoric acid under ×500 magnification

Evidence shows that application of phosphoric acid etching does not have the ability to enhance the bond strength of orthodontic brackets to composite surfaces. The reason is phosphoric acid cannot affect the organic phase and can only clean the composite surface (Fig. 2) [4, 15, 17, 18, 23]. Therefore, in the present study, unlike previous studies, 37% phosphoric acid etching process was considered for the control group and was applied as a basic step for all groups.

**Fig. 3:**
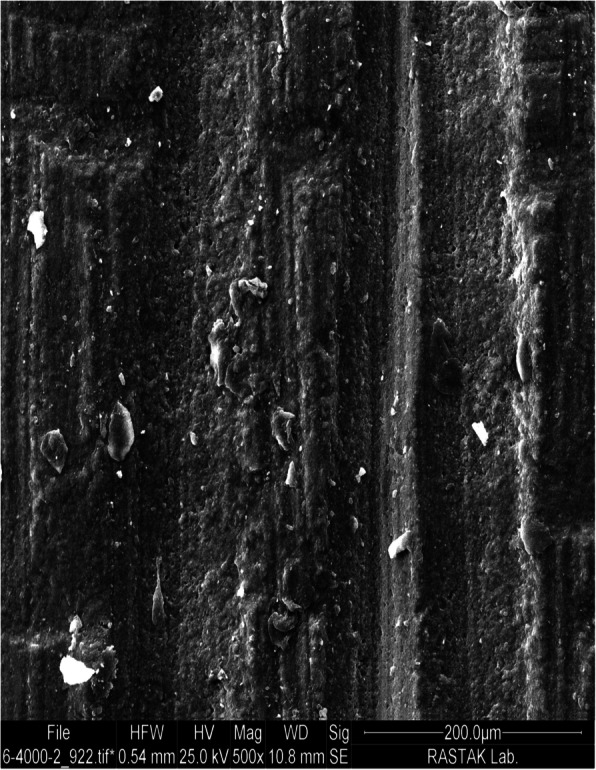
SEM micrograph of composite surface which has undergone grinding treatment under ×500 magnification

**Fig. 4:**
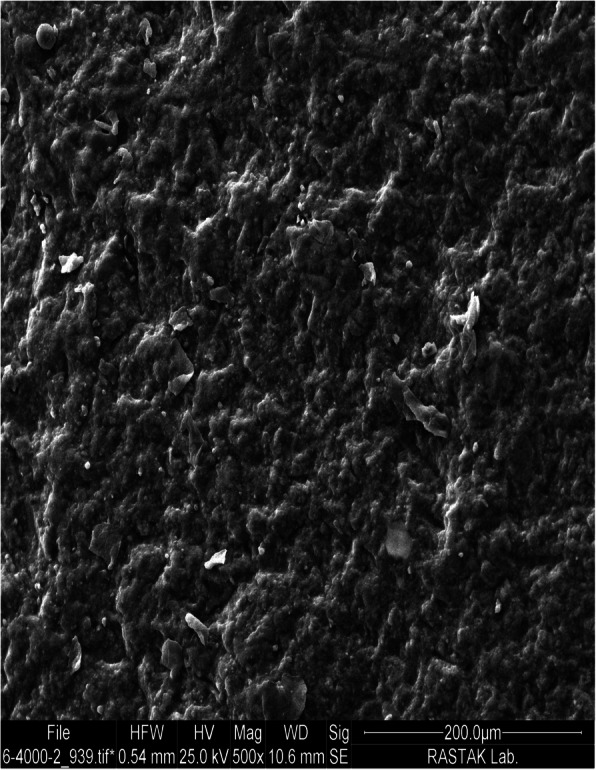
SEM micrograph of composite surface sandblasted with 50 μm aluminum oxide particles under ×500 magnification

The highest mean SBS value was obtained in the grinding group which was subjected to surface roughening by a diamond bur and high-speed handpiece, followed by the sandblasting group. Hammad and Banna [4] and Eslamian et al. [14] showed that grinding resulted in the highest mean SBS value among the study groups. This finding can be due to the fact that abrasive methods create deep craters and streaks in the composite surface, which result in high retention of orthodontic adhesive and can significantly enhance the mechanical interlocking and subsequently the SBS (Fig. 3) [4, 5, 13, 14, 24]. Some other studies indicated that sandblasting with Al_2_O_3_ particles resulted in the highest bond strength in comparison with other methods [15, 16]. Najafi et al. stated that sandblasting with Al_2_O_3_ particles can create microporosities on the composite surface and thus, increases the surface area for adhesive bonding (Fig. 4) [16]; while, diamond bur grinding creates both macro and micro-porosities on the bonding surface and therefore can be damaging for the composite surface [1], leading to a higher risk of plaque accumulation and subsequent caries development [18].

**Fig. 5:**
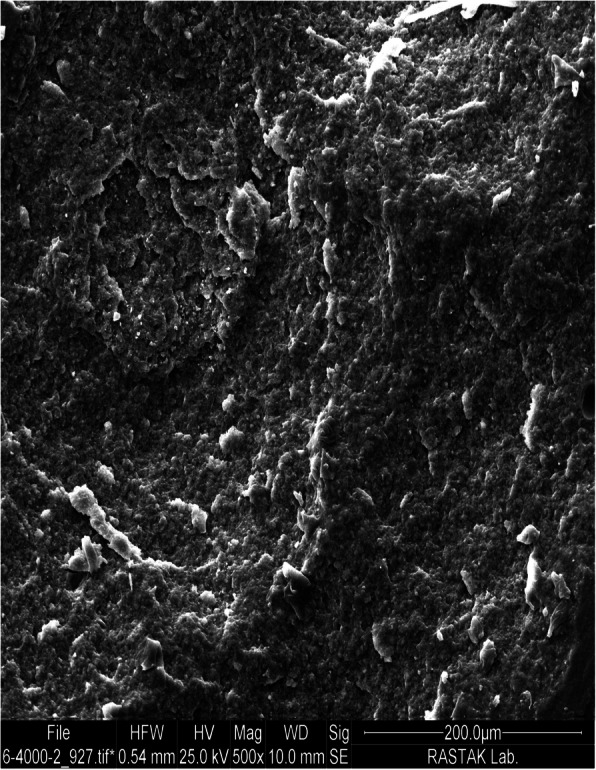
SEM micrograph of composite surface treated with Er,Cr:YSGG laser under ×500 magnification

Although the mean SBS difference between the Er,Cr:YSGG laser and the control groups was not statistically significant, application of laser increased the SBS in comparison with the control group and showed a clinically acceptable result since the SBS value of 6–8 MPa is considered clinically acceptable according to Reynold [25]. However, the control group did not show a clinically acceptable SBS. Improved SBS value in the laser group can be due to the ability of laser to provide micro retentive areas in the composite surface which increase the bonding surface area and thus, improve the bond strength of orthodontic brackets (Fig. 5) [18]. This result was similar to the findings of Korkmaz et al., who stated that Er,Cr:YSGG laser application resulted in a higher bond strength value than etching although the difference was not statistically significant [18]. Dehghani et al., also stated that Er:YAG laser was efficient for enhancing the bond strength between composite resin and orthodontic brackets, but the bond strength reported in the study by Dehghani et al. was higher than the value reported in our study [17]. It may be due to the difference in the type of composites and also the fact that their specimens, unlike the present study, were not subjected to aging process. In contrast, Sobouti et al. compared Er:YAG laser application (2 W and 3 W), bur abrasion, phosphoric acid etching, hydrofluoric acid etching, and sandblasting, and reported that 3 W laser application had the highest effect on the bond strength of metal brackets to aged composite [26]. The difference between their results and ours could be due to the difference in the laser types used in the two studies.

According to our findings, the most frequent ARI score in the grinding and sandblasting groups was score 3. This result indicates that the specimens in the abovementioned two groups had the most favorable adhesion between the composite surface and the adhesive. However, the disadvantage of this mode of bond failure is that it requires an additional step to remove the adhesive and polish the composite surface, which could be time consuming [16]. On the other hand, the ARI scores of laser and control groups were mostly 0 and 1. It could be stated that even though the adhesion in these two groups is not as great as that in the grinding and sandblasting groups, it would be quite easer to remove the remaining adhesive from the composite surface.

Given that the studies, done in this field so far, have presented a set of different methodology and results, the results of this study can work in favour of improving the knowledge in this field and consolidating previous studies with similar evidence. However, the present study had an in vitro design and the aging process was simulated. On the other hand, the current study evaluated Er,Cr:YSGG laser with one exposure setting; therefore, clinical studies are required on this topic and other settings of Er,Cr:YSGG laser irradiation.

## Conclusion

In conclusion, all groups except 37% phosphoric acid etching group showed clinically acceptable bond strength. Therefore, grinding, sandblasting, and Er,Cr:YSGG laser irradiation can be suggested as effective surface treatments for bonding of ceramic orthodontic brackets to aged composite.

## Data Availability

The datasets used and/or analyzed during the current study are available from the corresponding author on reasonable request.

## Abbreviations

SBS: Shear bond strength
ARI: Adhesive remnant index
SEM: Scanning electron microscopy
